# The Effect of Lower-Limb Exercise on Pain Management of the Patients Undergoing Posterior Lumbar Fusion Surgery: A Retrospective Case-Control Study

**DOI:** 10.1155/2021/3716696

**Published:** 2021-12-03

**Authors:** Tong Wu, Yong Ye

**Affiliations:** ^1^Department of Orthopedic Surgery, Nantong Tongzhou People's Hospital, 115 Jianshe Road, 226300 Nantong, Jiangsu, China; ^2^Department of Orthopedic Surgery, Nantong University Affiliated Tongzhou Hospital, 115 Jianshe Road, 226300 Nantong, Jiangsu, China; ^3^Department of Orthopedic Surgery, Wenzhou Central Hospital, 252 Baili East Road, 325000 Wenzhou, Zhejiang, China

## Abstract

**Purpose:**

The purpose of this study is to investigate the clinical effect of lower-limb exercise, when combined with celecoxib, on pain management of patients undergoing posterior lumbar fusion surgeries.

**Methods:**

The patients undergoing posterior lumbar fusion surgeries between 01/2018 and 06/2021 were retrospectively identified, with their data collected. After surgery, some patients took celecoxib for analgesia (celecoxib group, 200 mg/day) while the others took celecoxib together with lower-limb exercise (combined group, celecoxib-200 mg/day). On postoperative days (POD) 1, 3, 7, and 14, data were collected and analyzed regarding the following items: patient satisfaction, lower-limb muscle force, lumbar JOA score (29 points), Oswestry Disability Index (ODI), and visual analog scale (VAS) score.

**Results:**

A total of 225 participants were included in this study. Specifically, 120 cases were admitted into in the celecoxib group and 105 were included in the combined group. Comparisons of baseline data did not indicate any difference between the combined group and the celecoxib group. Data analysis showed that patient satisfaction in the combined group was significantly higher than the celecoxib group on POD 3, 7, and 14, respectively (all *p* < 0.001). Moreover, the combined group had less VAS score compared with the celecoxib group on POD 3, 7, and 14, respectively (all *p* < 0.01). In addition, lower-limb muscle force in the combined group was significantly stronger than that in the celecoxib group on POD 3 and POD 7, respectively (both *p* < 0.01). Furthermore, the combined group achieved less ODI score than the celecoxib group on POD 3, 7, and 14, respectively (all *p* < 0.05). Comparisons of the lumbar JOA score did not suggest any statistical difference during the whole follow-up period.

**Conclusions:**

In conclusion, postoperative lower-limb rehabilitation exercise can help to release pain after lumbar fusion surgeries. Additionally, postoperative lower-limb exercise can facilitate the recovery of lower-limb muscle force, as well as improving patient satisfaction.

## 1. Introduction

In clinical scenarios, lower back pain (LBP) mainly derives from intervertebral disc degeneration (IVDD) [1–3]. IVDD-related diseases, such as lumbar disc herniation, can lead to severe symptoms including LBP and lower-limb radicular pain. In such a situation, the patients usually need to undergo surgeries to remove the protruded disc and decompress the nerve root and spinal canal. To date, posterior lumbar interbody fusion surgery has been a widely used surgical procedure for treating IVDD-derived diseases, in particular, lumbar spinal diseases [4–6]. However, it has been reported by previous studies that postoperative patients may experience prolonged LBP and low quality of life [7–10], that is one of the key reasons that some patients would seek physical therapy after lumbar fusion surgery, for a purpose of speeding up their rehabilitation.

As suggested by previous studies, postoperative rehabilitation procedures can help pain management after spinal surgeries, even decreasing the disability events [11–13]. In the past few years, accumulative studies have indicated that postoperative lower-limb exercise can facilitate rehabilitation and help relieve pain after orthopedic surgery [14] and lumbar spine surgery [15, 16]. Clinically, celecoxib, one of nonsteroidal anti-inflammatory drugs (NSAIDS), has been commonly administered to the patients for pain relief, the regular oral dose of celecoxib being 200 mg daily [17–19]. However, it remains unclear whether postoperative lower-limb exercise can increase pain relief when administered together with celecoxib for the patients undergoing spine surgery.

Thus, the purpose of this study is to investigate the effect of lower-limb exercise, when combined with celecoxib, on pain management of the patients who undergo posterior lumbar fusion surgeries.

## 2. Patients and Methods

### 2.1. Ethics

Prior to the commencement of this study, the medical ethics has been approved by Medical Ethics Council of Nantong Tongzhou People's Hospital. All informed consent was signed by the patients (or their lawful guardians) before undergoing lumbar fusion surgeries.

### 2.2. Patients

**Figure 1:**
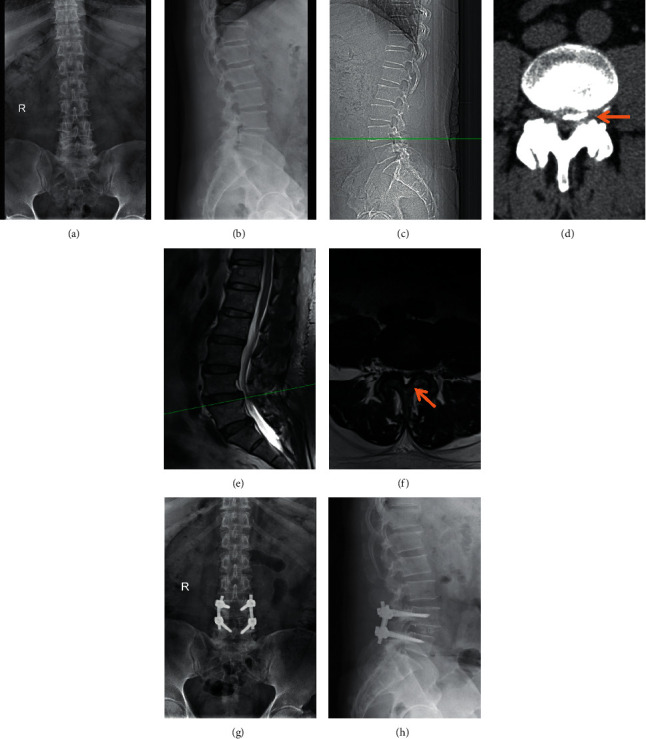
A representative case of posterior fusion surgery. (a)-(b) Preoperative X-ray radiographs. (c)-(d) Preoperative CT scan. (e)-(f) Preoperative MRI scan. (g)-(h) Postoperative X-ray radiographs. The arrows indicate the herniation of nucleus pulposus in the intervertebral disc.

The patients undergoing posterior lumbar fusion surgeries (Figure 1) between 01/2018 and 06/2021 were retrospectively identified and screened. All participants were diagnosed with lumbar disc herniation or lumbar spinal stenosis. The related data were then collected, including the data followed up with different time points on postoperative days (POD) 1, 3, 7, and 14. After surgery, some of these patients took celecoxib for analgesia (celecoxib group, celecoxib-200 mg/day), while the others took celecoxib together with lower-limb exercise (combined group, celecoxib-200 mg/day). Both in the celecoxib group and the combined group, celecoxib was administrated to the patients 200 mg/time/day, administrated in the evening. During the perioperation period, all the participants underwent the same routine medical care regardless of treatment groups. Postoperatively, the patients in the combined group did rehabilitation exercise by following the lower-limb rehabilitation procedures as previously reported [14–16] and maintained for up to 14 days.

### 2.3. Assessment

Data were collected and analyzed regarding the following items: patient satisfaction, lower-limb muscle force, lumbar JOA score (29 points), Oswestry Disability Index (ODI), and visual analog scale (VAS) score. The grading of lower-limb muscle force was based on the classification criteria which British Medical Research Council applies. In addition, the patient satisfaction rate was scored to three levels: very satisfied, satisfied, and dissatisfied.

### 2.4. Statistical Analyses

Statistical analysis in this study was performed using the software SPSS for Windows 18.0 (SPSS Inc., USA). The data of ODI, JOA score, and VAS score are presented as mean ± standard deviation (SD). The data of age are presented as median (range). Multiple comparisons were carried out with analysis of variance (ANOVA) if homogeneity and normality of variance were assumed, subsequently followed by Student–Newman–Keuls *t*-tests used to identify the difference between two groups. Moreover, chi-square tests were conducted to analyze the categorical data (gender, patient satisfaction, and muscle force). A *p* value of 0.05 was set as the significance level.

## 3. Results

### 3.1. Baseline Data of Participants

After the identification of all patients, a total of 225 participants were included in this case-control study. Specifically, 120 cases were admitted into the celecoxib group and 105 cases were included in the combined group. The combined group consists of 48 males and 57 females, while the celecoxib group consists of 54 males and 66 females. The median age of the combined group is 54 years (range 21–67), while the median age of the celecoxib group is 56 years (range 23–70). Comparisons of those baseline data did not suggest any difference between the combined group and the celecoxib group (all *p* > 0.05).

### 3.2. Patient Satisfaction

As given in Table 1, the patient satisfaction was categorized to three grades: very satisfied, satisfied, and dissatisfied. Most of the patients were very satisfied and satisfied about their treatment regardless of the treatment groups or postoperative time points. Data analysis showed that patient satisfaction in the combined group was significantly higher than the celecoxib group on POD 3, 7, and 14, respectively (all *p* < 0.001). There was no difference regarding the patient satisfaction on POD 1 between the combined group and the celecoxib group (*p* > 0.05).

### 3.3. VAS Score

As given in Table 2, the combined group obtained less VAS score compared with the celecoxib group on POD 3 (2.6 ± 1.2 vs. 3.5 ± 1.1), POD 7 (1.5 ± 1.2 vs. 2.3 ± 1.1), and POD 14 (1.1 ± 0.3 vs. 1.2 ± 0.2), respectively (all *p* < 0.01). No significant difference of VAS score was indicated on POD 1 between the combined group and the celecoxib group (*p* > 0.05).

### 3.4. Lower-Limb Muscle Strength Grading

As given in Table 3, most grading of the preoperative lower-limb muscle strength was grade III and grade IV in both the combined group and the celecoxib group. Grade IV and grade V (as a whole) took the majority after surgery, and grade V increased continuously during the postoperative follow-up period regardless of the groups. As compared with the preoperative grading, the muscle strength got improved in both the combined group and the celecoxib group. Lower-limb muscle force in the combined group was significantly stronger than that in the celecoxib group on POD 3 and POD 7, respectively (both *p* < 0.01). There was no significant difference found between the combined group and the celecoxib group on POD 1 or POD 14.

### 3.5. ODI Score

As given in Table 4, preoperatively, there was no significant difference regarding the ODI score between the combined group and the celecoxib group (*p* > 0.05). Postoperatively, both of the combined group and the celecoxib group achieved significant improvement of the ODI score, compared to their preoperative data, respectively. The combined group scored less ODI than the celecoxib group on POD 3, 7, and 14, respectively (all (*p* < 0.05)). There was no statistical difference between the combined group and the celecoxib group on POD 1 (*p* > 0.01).

### 3.6. Lumbar JOA Score

As given in Table 5, preoperatively, there was no significant difference regarding the lumbar JOA score between the combined group and the celecoxib group (*p* > 0.05). Postoperatively, comparisons of the lumbar JOA score did not suggest any statistical differences between the combined group and the celecoxib group during the whole follow-up period (*p* > 0.05).

## 4. Discussion

In our department, as a routine procedure for the prophylaxis of potential postoperative complications (such as deep vein thrombosis), the patients are required to do lower-limb rehabilitation exercise postoperatively; the exercise procedures are given in previous studies [14–16]. All participants are asked to do the same intensity rehabilitation for up to two weeks. The pain relief effect of lower-limb rehabilitation exercise on the patients after orthopedic surgery and spinal surgery has already been documented in those previous studies [14–16]. In this study, the patients in the combined group kept doing rehabilitation exercise for up to 14 days. That was because many patients can walk well and start to do some normal exercise, other than the lower-limb rehabilitation procedures, after 14 days after surgery. Thus, 14-day exercise with the lower-limb rehabilitation procedures after surgery was considered as an endpoint of our study on the postoperative pain management.

Celecoxib, a selective cyclooxygenase-2 inhibitor and an NSAID, has been routinely used by the patients after spinal surgery for pain relief, with its advantage of minimizing the gastrointestinal adverse effects [17]. Considering the pain relief effects of celecoxib and lower-limb exercise, it would be possible for them to have synergistic effects on pain relief. However, thus far, it has been unclear whether postoperative lower-limb exercise can increase pain relief when administered together with celecoxib for the patients undergoing spine surgery. Thus, this study was designed to investigate the effect of lower-limb exercise, when combined with celecoxib, on pain management of the patients who underwent posterior lumbar fusion surgery in our department.

As a result, a total of 225 cases were included in our study. Baseline data (age and gender) were well matched between the combined group and the celecoxib group. Compared with preoperative situations, the combined group and the celecoxib group have significantly improved in terms of the patient satisfaction, VAS score, lower-limb muscle force, lumbar JOA score, and ODI score. Also, it was found that the combined group achieved better results than the celecoxib group, in terms of the patient satisfaction, VAS score, lower-limb muscle force, and ODI score. These findings in this study stay consistent with the reports from previous studies [14–16] which indicate that lower-limb exercise can effectively increase postoperative pain relief, accelerate functional recovery, and decrease complications (such as deep vein thrombosis).

It is noticeable in this study that the postoperative lumbar JOA score is not significantly different between the combined group and the celecoxib group during the whole follow-up period (up to 14 days). This result of the JOA score is inconsistent with previous reports indicating that postoperative lower-limb exercise can improve the JOA score. One possible reason for this result is that our follow-up period is too short, only 14 days postsurgery. By contrast, the maximum follow-up period in the previous studies are up to 3 months [14–16]. Another reason could be the different study designs between this study and other studies. In this study, the combined group was designed to compare with the celecoxib group, while the lower-limb exercise group was compared with the control group (settings unknown) in those previous studies.

Up to now, there is no consensus regarding whether postoperative rehabilitation can effectively promote the recovery of patients undergoing spinal surgery. In terms of pain relief, functional improvement, and patient satisfaction, the positive effects of postoperative rehabilitation procedures have been declared in some studies [12, 14, 16, 20, 21], while some others are negative towards postoperative rehabilitation [4, 22, 23]. Apparently, the findings in the current study support the former, with increased pain relief, great functional improvement, and higher patient satisfaction in the rehabilitation group, compared to the nonrehabilitation group postoperatively.

This study has some limitations that might have restricted the interpretation of the data. First, this is a single-center, retrospective, case-control study, making the participants included lack for extensive representativeness and the data accuracy decreases to a certain extent. In addition, the patient sample is not large, just a total of 225 participants were included in this study. It would make the results and conclusions more robust if the patient sample size is greater. Moreover, the follow-up period in this study is not long enough (just 14 days), which can potentially influence the results and conclusions. Therefore, a better future study needs to resolve all of the shortcomings listed above. It can be designed to be multicenter, prospective, blinded, and randomly controlled; the sample size should be big enough.

## 5. Conclusions

In summary, postoperative lower-limb rehabilitation exercise can synergistically work with celecoxib, increasing pain relief for the patients undergoing lumbar fusion surgeries. In addition, postoperative lower-limb exercise can facilitate functional recovery and increase patient satisfaction.

## Figures and Tables

**Table 1:** Patient satisfaction at discharge.

After surgery	Combined group (*n* = 105)	Celecoxib group (*n* = 120)	Chi-square tests	
	Very satisfied/satisfied/dissatisfied	Very satisfied/satisfied/dissatisfied	*χ* ^2^	*P* value
POD 1	30 cases/60 cases/15 cases	32 cases/69 cases/19 cases	0.164	0.921
POD 3	72 cases/30 cases/3 cases	40 cases/64 cases/16 cases	**29.47**	<**0.001**
POD 7	92 cases/12 cases/1 cases	52 cases/58 cases/10 cases	**47.92**	<**0.001**
POD 14	101 cases/4 cases/0 case	78 cases/37 cases/5 cases	**33.67**	<**0.001**

All values with P < 0.05 are presented in bold, which indicate statistical significance. Combined group, celecoxib and lower-limb rehabilitation exercise; POD, postoperative day.

**Table 2:** Assessment of VAS score.

Group	Pre-op	POD 1	POD 3	POD 7	POD 14
Celecoxib (*n* = 120)	6.8 ± 1.7	5.7 ± 1.4	3.5 ± 1.1	2.3 ± 1.1	1.2 ± 0.2
Combined (*n* = 105)	6.6 ± 2.1	5.4 ± 1.6	2.6 ± 1.2	1.5 ± 1.2	1.1 ± 0.3
*P* value	0.431	0.135	<**0.001**	<**0.001**	**0.003**

All values with P < 0.05 are presented in bold, which indicate statistical significance.VAS, visual analog scale; combined group, celecoxib and lower-limb rehabilitation exercise; POD, postoperative day; Pre-op, preoperation.

**Table 3:** Lower-limb muscle force.

Group	Pre-op			POD 1			POD 3			POD 7			POD 14		
Grade	III	IV	V	III	IV	V	III	IV	V	III	IV	V	III	IV	V
Celecoxib (*n* = 120)	34	68	18	31	77	12	28	75	17	25	64	31	12	24	84
Combined (*n* = 105)	27	63	15	23	72	10	18	52	35	12	43	50	8	18	79
*χ* ^2^	0.268			0.537			11.622			12.200			0.814		
*P* value	*P*=0.875			*P*=0.764			*P*=0.003			*P*=0.002			*P*=0.666		

*P* < 0.001, in terms of muscle force comparison between the celecoxib group and the combined group. POD, postoperative day; combined group, celecoxib and lower-limb rehabilitation exercise; Pre-op, preoperation.

**Table 4:** ODI assessment and comparisons.

Groups	Pre-op	POD 1	POD 3	POD 7	POD 14
Celecoxib (*n* = 120)	48 ± 22	41 ± 20	33 ± 16	21 ± 11	13 ± 4
Combined (*n* = 105)	46 ± 23	40 ± 19	27 ± 15	18 ± 9	11 ± 4
*P* value	0.506	0.702	**0.0042**	**0.027**	<**0.001**

All values with P < 0.05 are presented in bold, which indicate statistical significance.ODI, Oswestry Disability Index; combined group, celecoxib and lower-limb rehabilitation exercise; POD, postoperative day; Pre-op, preoperation.

**Table 5:** JOA score (lumbar, 29 points) assessment and comparisons.

Groups	Pre-op	POD 1	POD 3	POD 7	POD 14
Celecoxib (*n* = 120)	7.5 ± 1.3	10.5 ± 1.5	14.7 ± 2.2	19.8 ± 6.1	22.1 ± 6.6
Combined (*n* = 105)	7.8 ± 1.2	10.2 ± 1.7	15.1 ± 2.4	20.3 ± 6.3	22.3 ± 6.5
*P* value	0.075	0.161	0.194	0.546	0.820

JOA, Japanese Orthopedic Association; combined group, celecoxib and lower-limb rehabilitation exercise; POD, postoperative day; Pre-op, preoperation.

## Data Availability

The data used to support the findings of this study are available from the corresponding author upon request.
